# Understanding factors associated with the trajectory of subjective cognitive complaints in groups with similar objective cognitive trajectories

**DOI:** 10.1186/s13195-023-01348-w

**Published:** 2023-11-22

**Authors:** Federica Cacciamani, Ariane Bercu, Vincent Bouteloup, Leslie Grasset, Vincent Planche, Geneviève Chêne, Carole Dufouil, for the MEMENTO Cohort Study Group

**Affiliations:** 1 UMR 1219, Bordeaux Population Health Center, University of Bordeaux, Inserm, Bordeaux, F-33000 France; 2 CIC 1401-EC, Inserm, University of Bordeaux, CHU de Bordeaux, F-33000 Bordeaux, France; 3 ARAMISLab, Sorbonne Université, Institut du Cerveau - Paris Brain Institute - ICM, CNRS, Inria, Inserm, AP-HP, Hôpital de la Pitié Salpêtrière, Paris, F-75013 France; 4 Qairnel SAS, Paris, France; 5 Department of Public Health, CHU de Bordeaux, 33000 Bordeaux, France; 6 CHU de Bordeaux, Pôle de Neurosciences Cliniques, Centre Mémoire de Ressources Et de Recherche, 33000 Bordeaux, France; 7 University of Bordeaux, CNRS UMR 5293, Institut Des Maladies Neurodégénératives, 33000 Bordeaux, France

**Keywords:** Cognitive complaints, Objective cognitive trajectories, Depression, Comorbidity-polypharmacy, Loneliness, Blood-based AD biomarkers, Alzheimer’s disease, Dementia risk, Longitudinal study, Cohort study, Latent class mixed model

## Abstract

**Background:**

Cognitive complaints are often regarded as an early sign of Alzheimer’s disease (AD) but may also occur in several other conditions and contexts. This study examines the correlates of cognitive complaint trajectories over a 5-year period in individuals who shared similar objective cognitive trajectories.

**Methods:**

We analyzed a subsample (*n* = 1748) of the MEMENTO cohort, consisting of individuals with subjective cognitive decline or mild cognitive impairment at baseline. Participants were stratified based on their latent MMSE trajectory over a 5-year period: “high and increasing,” “subtle decline,” and “steep decline.” Within each of the three strata, we used a latent-class longitudinal approach to identify distinct trajectories of cognitive complaints. We then used multiple logistic regressions to examine the association between these complaint trajectories and several factors, including AD biomarkers (blood pTau/Aβ42 ratio, cortical thickness, APOE genotype), anxiety, depression, social relationships, a comorbidity-polypharmacy score, and demographic characteristics.

**Results:**

Among participants with high and increasing MMSE scores, greater baseline comorbidity-polypharmacy scores (odds ratio (OR) = 1.30, adjusted *p* = 0.03) were associated with higher odds of moderate and increasing cognitive complaints (as opposed to mild and decreasing complaints). Baseline depression and social relationships also showed significant associations with the complaint pattern but did not survive correction for multiple comparisons. Among participants with subtle decline in MMSE scores, greater baseline depression (OR = 1.76, adjusted *p* = 0.02) was associated with higher odds of moderate and increasing cognitive complaints (versus mild and decreasing). Similarly, baseline comorbidity-polypharmacy scores and pTau/Aβ_42_ ratio exhibited significant associations, but they did not survive correction. Among participants with a steep decline in MMSE scores, greater baseline comorbidity-polypharmacy scores increased the odds of moderate complaints (versus mild, OR = 1.38, unadjusted *p* = 0.03, adjusted *p* = 0.32), but this effect did not survive correction for multiple comparisons.

**Conclusions:**

Despite similar objective cognitive trajectory, there is heterogeneity in the subjective perception of these cognitive changes. This perception was explained by both AD-related and, more robustly, non-AD-related factors. These findings deepen our understanding of the multifaceted nature of subjective cognitive complaints in individuals at risk for dementia and underscore the importance of considering a range of factors when interpreting cognitive complaints.

**Supplementary Information:**

The online version contains supplementary material available at 10.1186/s13195-023-01348-w.

## Background

Cognitive decline associated with advanced age has emerged as a major public health issue, with Alzheimer’s disease (AD) standing out as the prevailing cause [1, 2]. As a result, an increasing number of individuals are concerned about their cognitive health, even in the absence of objective cognitive impairment [3, 4]. Assessment of cognitive complaints could provide important clues to possible progressive cognitive decline and prompt additional testing to determine the underlying cause [5]. A cognitive complaint is a person’s report of his or her own cognitive problems, such as those related to attention, memory, or language.

While cognitive complaints are commonly considered an early sign of AD [6–9], it is important to recognize that they can also occur in several other conditions and contexts. Cognitive complaints are frequently reported in the elderly population [10], suggesting that their occurrence cannot be attributed solely to the presence of AD. Moreover, in cohorts of individuals presenting to a memory clinic for clinical evaluation for cognitive problems, the association between the severity of cognitive complaints and AD biomarkers or cognitive scores is not consistently observed [11]. This highlights the need to consider factors and conditions other than AD that may contribute to cognitive complaints.

Anxiety, depression, perceived loneliness, and certain conditions (such as diabetes, cardiovascular disease, and sleep disorders) may exacerbate the perception of cognitive decline, which may not be detected or detected to a lesser degree on testing [12–16]. It is also important to note that other factors can contribute to individuals expressing fewer cognitive complaints compared to what is objectively observed through tests. These factors encompass cognitive bias, fear of stigmatization, fear of losing independence, and the presence of symptoms such as anosognosia, which is common in many neurological conditions, including AD. Anosognosia, specifically, may prevent individuals with cognitive impairment from accurately reporting their cognitive deficits, due to a lack of awareness regarding the severity or impact of their impairment [17, 18]. Because of the interplay of all these factors, there is not always a direct correspondence between self-perceived changes in cognitive ability and actual changes, as measured by objective tests or by external evaluations [19, 20].

Using data from a large clinic-based study of individuals at risk for dementia, we aimed to identify the main trajectories of subjective cognitive complaints over a 5-year period in groups of individuals sharing similar objective cognitive changes. We then aimed to examine the correlates of these identified trajectories of cognitive complaints.

## Methods

### Participants

MEMENTO is a French multicentric prospective cohort study. Detailed study procedures were previously published in [21]. The criteria for inclusion in MEMENTO were: age 18 or older, Clinical Dementia Rating (CDR) score of 0 or 0.5, and either subjective cognitive decline (SCD) or a recent diagnosis of MCI. Individuals with SCD had to be at least 60 years of age to participate in the study.

Of 2449 individuals screened at 26 French university memory clinics between 2011 and 2014, a total of 2323 consented to participate in the study. Participants were followed up at least annually for up to 5 years.

Each annual visit included a complete clinical and psychosocial assessment. At baseline, 2 years follow-up, and 4 years follow-up, blood sampling and brain MRI were undertaken following standardized procedures.

### Measures

#### Cognitive complaints

Cognitive complaints were assessed using a visual analog scale. Participants were asked to indicate whether they experienced difficulties in memory (learning new things, remembering recent events), attention (concentrating, dividing their attention between more than one task, remembering what they were going to do or say when disturbed), and language (word-finding, writing, or reading). All three dimensions were rated from 0 (not at all) to 10 (very much). The complaint score was computed as the sum of the responses to the three dimensions and thus ranged from 0 (no complaint) to 30 (severe complaint). The scale was completed every 6 to 12 months.

#### Objective cognitive performance

We used the Mini-Mental Score Examination (MMSE) as a global measure of objective cognitive performance [22]. The scale consists of 30 questions with a response option of 0/1, with a higher score representing better cognitive performance. The MMSE was administered every 6 months.

#### Anxiety and depression symptoms

To measure anxiety and depression, we used the Neuropsychiatric Inventory (NPI), a validated instrument specifically designed to assess neuropsychiatric symptoms in various neurological disorders, including dementia [23]. The NPI anxiety score provides a quantitative assessment of anxiety-related symptoms, which include excessive worry, agitation, tension, and feelings of apprehension. The anxiety score was derived from 14 items. Each item was scored on a scale of 0 to 3 (total score ranges from 0 to 42), with higher scores indicating higher levels of anxiety. The NPI depression score assesses depression-related symptoms such as sadness, hopelessness, and loss of interest or pleasure. The depression score was derived from 13 items. Each item was scored on a scale of 0 to 3 (total score of 0 to 39), with higher scores indicating greater symptoms of depression.

#### Comorbidity-polypharmacy score

The comorbidity-polypharmacy score, previously validated for assessing comorbidity burden in the context of brain injury [24], was calculated as the sum of the individual’s known diagnoses and the number of medications he or she was taking at the time of the visit. This scoring system allows quantification of the overall severity of comorbid conditions, with polypharmacy serving as an indicator of the severity of the condition(s) affecting the individual.

#### Social relationships

To assess the social relationships and social support experienced by participants, we employed a subjective measure. Participants were asked to indicate, without any restrictions on the type of relationship, the number of individuals to whom they felt emotionally close. This included family members, friends, work colleagues, or any other meaningful connections in their lives. Specifically, participants were requested to provide a numerical count of the individuals within their social network with whom they shared a sense of closeness or connection.

#### Blood pTau/Aβ_42_ ratio

We considered the ratio between blood levels of threonine_181_ pTau (pTau_181_) and Aβ_42_. A higher pTau/Aβ_42_ ratio in blood indicates a higher level of AD pathology [25].

Both baseline pTau_181_ and Aβ_42_ were measured using Simoa HD-X technology and commercial kits on a Quanterix HD-X analyzer: Plasma Aβ42 using Neurology 3-Plex A Advantage Kit (item No. 101995) and serum p181-tau using p181-tau Advantage V2 Kit (item No. 103714). All the datasheets and validation reports are available on the manufacturer’s website (quanterix.com/simoa-assay-kits/). Measurements were performed in the research platform of the University Hospital of Bordeaux where the centralized biobank is stored and blinded from all other data.

#### Cortical thickness

As a structural biomarker for AD, we used the average cortical thickness on a T1-weighted MRI of the AD signature regions: bilateral entorhinal cortex, inferior temporal cortex, middle temporal cortex, inferior parietal cortex, fusiform cortex, and precuneus [26]. Cortical thickness was measured by the CATI group (cati-neuroimaging.com) using Freesurfer. The pipeline was described elsewhere [27]. White matter and pial surfaces were extracted and aligned using a 2D template to achieve point-to-point correspondence. Cortical thickness was defined as the distance between the white matter and pial surfaces at more than 100,000 locations. Visual quality control was performed using 3D visualization of the surface models. Subsequently, the Desikan atlas was used to divide the cortical surface into 34 regions of interest (ROIs) on both hemispheres, resulting in a total of 68 ROIs [28]. The measurements are given in cm^3^.

#### APOE genotype

As a genetic risk factor for AD, we used the apolipoprotein E (APOE) genotype dichotomized into ε4 allele carriers (at least one ε4 allele) and non-carriers (no ε4 alleles).

#### Ascertainment of dementia cases

From month 6 to month 60, participants were considered to have possibly reached the clinical stage of dementia if they had a cognitive decline and/or behavioral deficits severe enough to interfere with their social life or daily autonomy, based on the criteria of DSM-IV-TR and NINCDS-ADRDA [29, 30]. Neurology/geriatrics specialists reviewed the available data and classified dementia cases into subtypes using standardized criteria. To ensure the accuracy of dementia diagnoses, clinical dementia diagnoses were reported to the coordinating center. The dementia validation panel then submitted its diagnoses within 2 weeks and reached a consensus through agreement or structured telephone meetings. An annual face-to-face meeting was held for quality control and review of selected cases. The incidence rate of dementia was calculated by dividing the number of new cases observed during the 60-month study period by the sum of the time that each participant was under observation and considered at risk of developing dementia. This incidence rate was expressed as cases per 1000 person-years.

### Statistical analyses

#### Identification of objective cognitive trajectories (MMSE) and sample stratification

We used the *NormPsy* package in R 4.2.2 to transform the MMSE raw scores into normalized scores between 0 and 100. During normalization, the ranking of the score is preserved, but the distances between two consecutive values are transformed to correct for curvilinearity. This normalization is specifically tailored to heterogeneous populations including older individuals with normal and pathological cognitive performance [31].

We then identified an optimal number of latent classes of individuals sharing similar MMSE trajectories. For this purpose, we performed latent class mixed models (LCMMs) using the *lcmm* R package [32, 33]. Normalized MMSE scores were explained by a quadratic function of time since inclusion (measured in years) at the population level (fixed effects). To account for individual-level variation, we considered both linear models with only random intercepts and more complex models with random effects on both the intercept and the slope of the quadratic function. Our model selection process, which included a grid search with different model structures, eventually led to the selection of the final model. To determine the optimal number of latent classes, we iteratively adjusted the number of classes while maintaining the same model structure. We performed 50 repetitions of model fitting for each number of classes. Model selection was based on the Bayesian information criterion (BIC), with the lowest BIC value indicating the selected number of classes. The LCMMs were performed on 11,339 observations and the 3-class model had the lowest BIC. All three mean posterior probabilities of class membership were > 70%. Homoscedasticity and normality of the residuals were checked visually. For each model, we tested 50 sets of random initial values to reduce the probability of convergence to a local maximum. Parameter significance is evaluated by multiple or simple Wald test with significance level *α* = 0.05. We then divided the sample into strata based on the latent classes obtained, each representing a trajectory of the MMSE. The next steps of the analysis were conducted separately within each stratum.

#### Identification of trajectories of cognitive complaint in each stratum

Within each stratum of objective cognitive trajectory (MMSE), we identified an optimal number of trajectories of cognitive complaints. We used the same approach as described above but with the complaint scores as the dependent variable. Within each stratum, the 2-class model had the lowest BIC and was therefore selected as the best model. In other words, we identified two distinct latent classes within each stratum, each exhibiting a specific trajectory of cognitive complaint over a 5-year period. For all latent classes, the mean posterior probability of class membership was > 70%.

#### Factors associated with cognitive complaint trajectories in each stratum

We used logistic regression models to examine factors associated with the trajectory of cognitive complaints. Specifically, we examined the relationship between the dependent variable, i.e., the trajectory of cognitive complaints, and multiple independent variables, including baseline depression, anxiety, comorbidity-polypharmacy score, social network, AD biomarkers, and demographic variables (age, sex, and education). We estimated the odds of exhibiting a particular trajectory of cognitive complaints based on these variables. All numeric independent variables were centered and scaled in order to make the coefficients comparable. The collinearity of independent variables was checked using Pearson’s correlations and the variance inflation factor (VIF; evidence of collinearity if VIF > 2). To address the issue of multiple comparisons, we applied false discovery rate (FDR) correction to control for the expected proportion of false positives among statistically significant results. We used a critical FDR threshold of 0.05 to adjust the *p*-values obtained from our logistic regression models.

## Results

### Description of the sample

**Fig. 1:**
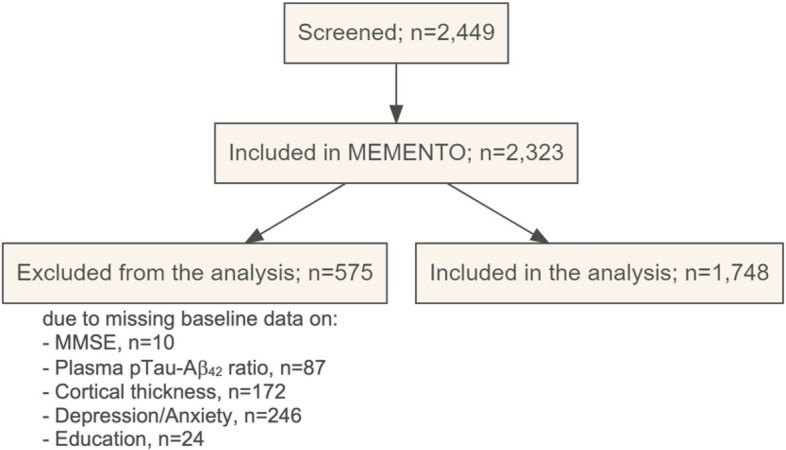
Flow diagram illustrating participant selection from the MEMENTO cohort

Our analytical sample consisted of 1748 participants (Fig. 1) who had complete data for our variables of interest (baseline MMSE, complaint, NPI anxiety and depression, comorbidity-polypharmacy score, social network, pTau/Aβ_42_ ratio, cortical thickness, APOE genotype, age, sex, and education).

Our analytical sample was 62.4% female (*n* = 1091), the mean age at inclusion was 70.5 years (standard deviation, SD = 8.57), and 65.2% (*n* = 1140) had a high school diploma or higher education. At inclusion, the mean MMSE score was 27.9 (SD = 1.90), and 40.8% had a CDR of 0 (vs 0.5 for the others). The median number of available visits per subject was 10 (interquartile range of 7 to 11). 96.2% of the sample had at least two MMSE scores available and 95.1% had at least two complaint scores available.

### Sample stratification based on the trajectory of the MMSE

Stratum #1 (“high baseline and slightly increasing MMSE”) included 1044 participants (59.7% of the sample) with a mean normalized MMSE of 85.11 out of 100 at baseline (SD = 11.55) which increased significantly by 0.93 points per year on average (95%CI = 0.24–1.73, *p*-value < 0.01). At baseline, 51.3% had CDR = 0 (vs. CDR = 0.5 for the others). The dementia incidence rate in this stratum was 3.46 per 1000 person-years.

Stratum #2 (“Subtle decline in MMSE scores”) included 445 participants (25.5% of the sample) with a mean normalized MMSE of 71.41 at baseline (SD = 11.13), which remained mostly stable on average for the first 2.5 years and then began to decline, reaching a mean normalized MMSE of 63.11 at 5 years (*p*-value < 0.01). At baseline, 63.6% had CDR = 0.5 (vs. CDR = 0 for the others). The dementia incidence rate in this stratum was 22.84 per 1000 person-years.

Stratum #3 (“Steep decline in MMSE scores”) included 256 participants (14.8% of the sample) with a mean normalized MMSE of 60.15 at baseline (SD = 13.35) which decreased significantly by an average of 6 points per year (*p*-value < 0.01). At baseline, 93% had CDR = 0.5 (vs. CDR = 0 for the others). The dementia incidence rate in this stratum was 260.75 per 1000 person-years.

**Fig. 2:**
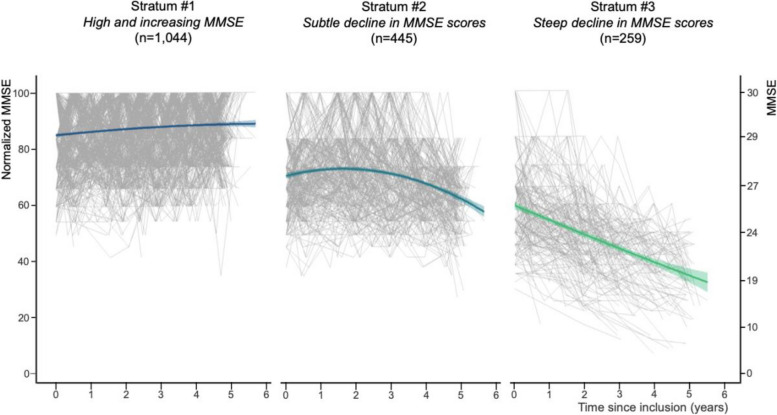
Trajectories of the MMSE in our subsample of MEMENTO study participants. *Note*. The trajectories of the MMSE were estimated using a longitudinal latent class approach

The characteristics of participants within the three strata are described in Table 1. Figure 2 shows the MMSE trajectory in the three strata.

**Table 1:** Characteristics of the individuals within the three strata of objective cognitive trajectory in our subsample of MEMENTO study participants

	**Stratum #1**	**Stratum #2**	**Stratum #3**
Description	High and increasing MMSE	Subtle decline in MMSE scores	Steep decline in MMSE scores
*N* (% of the sample)	1044 (59.7)	445 (25.5)	259 (14.8)
Normalized MMSE at baseline [/100; mean (SD)]	85.11 (11.55)	71.41 (11.13)	60.15 (13.35)
Age at baseline [years; mean (SD)]	69.51 (8.46)	71.11 (8.63)	73.77 (7.96)
Sex [female; *n* (%)]	668 (64.0)	276 (61.9)	148 (57.1)
Education [low; *n* (%)]	261 (25.0)	205 (56.0)	143 (55.2)
Clinical Dementia Rating at baseline [0.5; *n* (%)]	509 (48.7)	283 (63.6)	241 (93.0)
Dementia incidence rate [per 1000 person-years]	3.46	22.84	260.75

*Note*. Numerical variables are described by means and SD (standard deviations). Categorical variables by counts and percentages. Low education, less than a high school diploma

### Trajectories of cognitive complaints within strata of objective cognitive trajectory

**Fig. 3:**
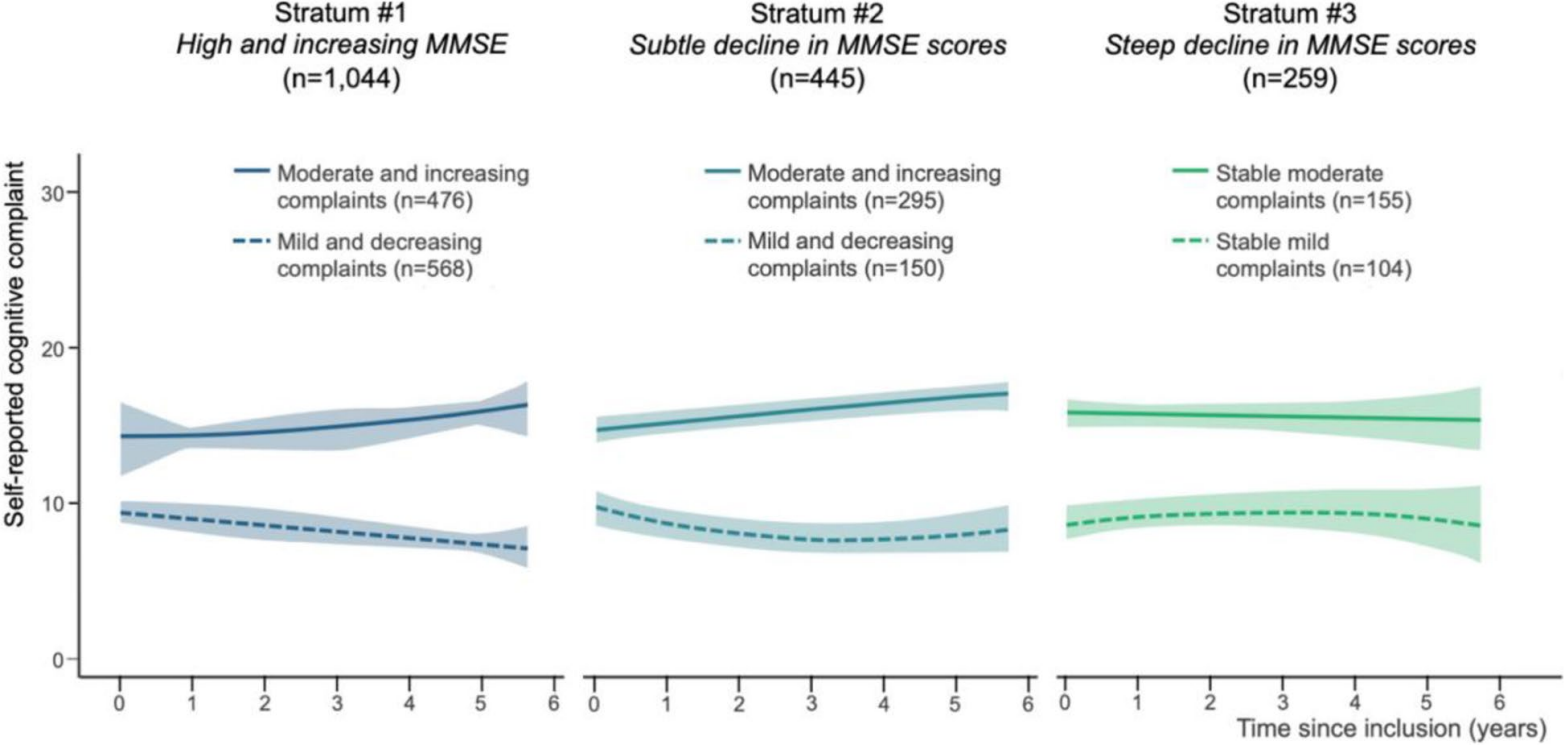
Trajectories of subjective cognitive complaints stratified by objective cognitive trajectory, in our subsample of MEMENTO study participants. *Note*. In each stratum, the trajectories of cognitive complaints were estimated using a longitudinal latent class approach

The trajectories of cognitive complaints for each stratum of objective cognitive trajectory are in Fig. 3. Their description is in Table 2.

**Table 2:** Description of the latent classes of subjective cognitive complaints stratified by objective cognitive trajectory, in our subsample of MEMENTO study participants

Stratum	**Stratum #1** ***High and increasing MMSE* (*n* = 1044)**		**Stratum #2** ***Subtle decline in MMSE scores* (*n* = 445)**		**Stratum #3** ***Steep decrease in MMSE scores* (*n* = 259)**	
Cognitive complaint trajectory	**Moderate and increasing complaint**	**Mild and decreasing complaint**	**Moderate and increasing complaint**	**Mild and decreasing complaint**	**Stable moderate complaint**	**Stable mild complaint**
*N* (% of the stratum)	476 (45.6)	568 (54.4)	295 (66.4)	150 (33.6)	155 (59.8)	104 (40.1)
Cognitive complaint score at baseline [/30; mean (SD)]	15.04 (5.40)	9.13 (5.03)	15.02 (5.33)	9.00 (5.10)	16.15 (4.27)	7.79 (4.11)
Age at baseline [years; mean (SD)]	68.99 (8.91)	69.96 (8.04)	70.78 (8.94)	71.75 (7.98)	73.13 (8.26)	74.73 (7.43)
Sex [female; *n* (%)]	312 (65.5)	356 (62.7)	186 (63.1)	89 (59.3)	95 (61.3)	53 (51.0)
Education [low; *n* (%)]	118 (24.8)	143 (25.2)	140 (47.5)	64 (42.7)	82 (52.9)	61 (58.7)
Dementia incidence rate [per 1000 person-years]	2.82	3.99	22.29	23.98	231.06	308.67
Blood pTau/Aβ_42_ at baseline [pg/mL; mean (SD)]	0.10 (0.15)	0.10 (0.29)	0.12 (0.15)	0.09 (0.07)	0.15 (0.12)	0.20 (0.29)
Cortical thickness at baseline [cm^3^; mean (SD)]	2.35 (0.10)	2.34 (0.09)	2.33 (0.10)	2.32 (0.09)	2.28 (0.09)	2.27 (0.10)
APOE genotype [noncarrier; *n* (%)]	363 (76.3)	432 (76.1)	201 (68.1)	47 (31.3)	78 (50.3)	52 (50.0)
Comorbidity-polypharmacy at baseline [mean (SD)]	7.24 (5.03)	6.24 (4.11)	8.53 (4.95)	7.03 (4.78)	7.46 (4.33)	6.51 (4.55)
Depression score at baseline [mean (SD)]	1.61 (2.87)	0.84 (2.19)	2.20 (3.70)	0.65 (1.94)	1.43 (2.70)	2.08 (3.33)
Anxiety score at baseline [mean (SD)]	2.01 (3.37)	1.26 (2.82)	2.32 (3.22)	1.28 (2.72)	2.01 (3.15)	2.69 (4.35)
Social network [Nb of close relationships; mean (SD)]	5.91 (4.93)	6.78 (5.76)	5.77 (5.51)	6.53 (9.23)	6.47 (5.68)	7.13 (7.02)

*Note.* Numerical variables are described by means and SD (standard deviations). Categorical variables by counts and percentages. Low education: less than a high school diploma; pTau/Aβ42, ratio between levels of threonine181 pTau and β-amyloid42; APOE, apolipoprotein E

In participants presenting with high baseline and slightly increasing MMSE scores (*n* = 1044), 45.6% (*n* = 476) reported moderate cognitive complaints at baseline (mean = 15.04, SD = 5.40), which increased in intensity to a mean of 16.42 (SD = 3.82) at the 5-year visit. Conversely, 54.4% (*n* = 568) reported mild complaints at baseline (mean = 9.13, SD = 5.03), which decreased to a mean of 7.19 (SD = 3.75) after 5 years. In both latent classes, the change over time was found to be significant (both *p*-value < 0.01).

In participants who showed a subtle decline in MMSE scores (*n* = 445), 66.29% (*n* = 295) reported moderate cognitive complaints at baseline (mean = 15.02, SD = 5.33), which increased in intensity to a mean of 16.81 (SD = 4.41) at the 5-year visit. Conversely, 33.71% (*n* = 150) reported mild complaints at baseline (mean = 9.00, SD = 5.10), which decreased to a mean of 7.14 after 5 years (SD = 3.97). In both latent classes, the change over time was found to be significant (both *p*-value < 0.01).

In participants who showed a steep decline in MMSE scores (*n* = 259), 59.8% (*n* = 155) reported moderate cognitive complaints at baseline (mean = 16.15, SD = 4.27), while 40.2% (*n* = 104) reported mild complaints at baseline (mean = 7.79, SD = 4.11). During the follow-up period, there was no significant change in the intensity of complaints in both classes (first class: *p*-value = 0.90; second class: *p*-value = 0.44).

### Characterization of complaint trajectories

**Fig. 4:**
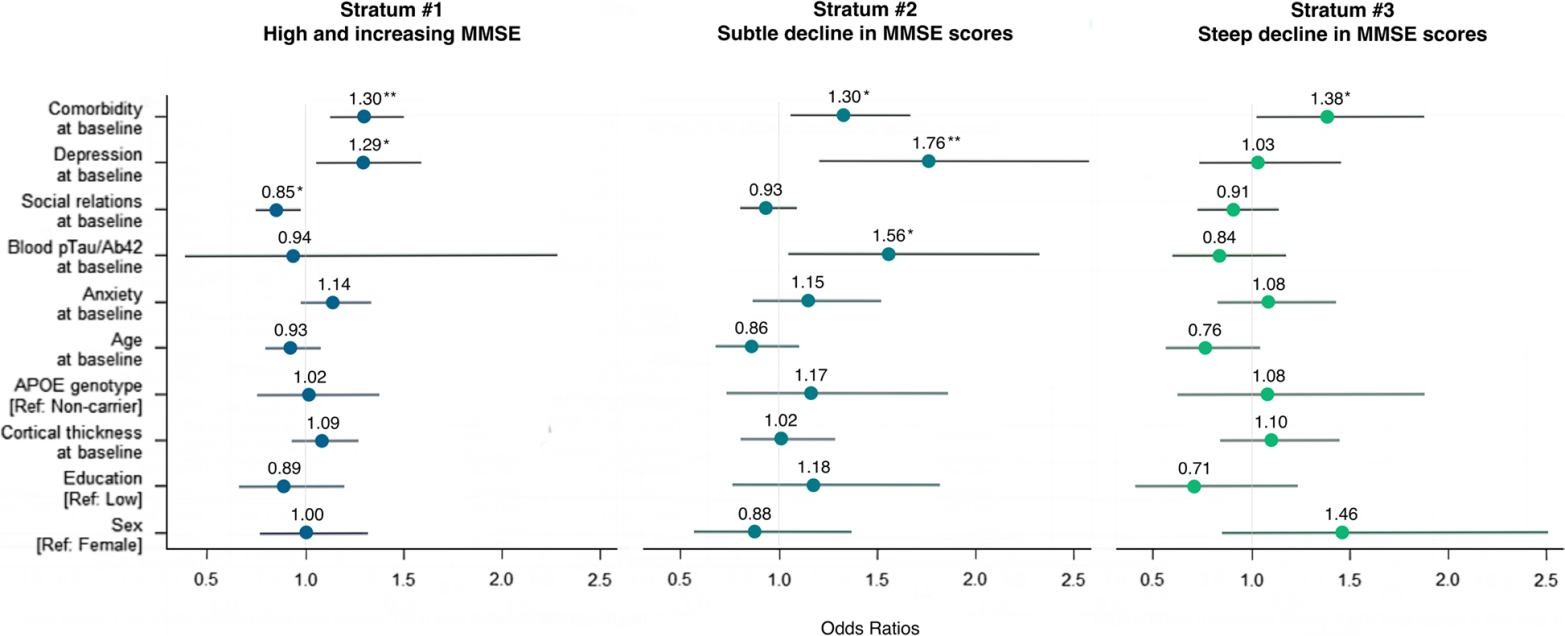
Results from multivariable logistic regression modeling the association between subjective complaint trajectory and the variables of interest, stratified by objective cognitive trajectory, in our subsample of MEMENTO study participants. *Note*. The dependent variable is the cognitive complaint trajectory. The reference category is the “mild and decreasing complaint” trajectory for Strata #1 and #2, and the “mild complaint” trajectory for Stratum #3. *Statistically significant before FDR correction (i.e., *p* < 0.05). **Statistically significant after FDR correction (i.e., *p* < 0.05)

Figure 4 shows the associations of baseline depression, anxiety, comorbidity-polypharmacy score, social relationships, AD biomarkers, and demographic variables with cognitive complaint trajectory, stratified by objective cognitive trajectory.

In participants with high baseline and slightly increasing MMSE (Stratum #1), greater comorbidity-polypharmacy scores (OR = 1.30, 95%CI = 1.10–1.54, unadjusted *p* < 0.01, adjusted *p* = 0.03) were associated with significantly increased odds of moderate and increasing complaints (versus mild and decreasing). Greater depression symptoms (OR = 1.29, 95%CI = 0.98–1.71, unadjusted *p* = 0.01, adjusted *p* = 0.07) and fewer close social relationships (OR = 0.85, 95%CI = 0.74–0.98, unadjusted *p* = 0.02, adjusted *p* = 0.08) were also associated with significantly increased odds of moderate and increasing complaints (versus mild and decreasing), but these associations did not survive FDR correction. Within this stratum, anxiety, baseline AD biomarkers, nor demographic variables were associated with the trajectory of cognitive complaints (all unadjusted *p* > 0.09, all adjusted *p* > 0.26).

In participants who showed a subtle decline in MMSE scores (Stratum #2), greater depression symptoms (OR = 1.76, 95%CI = 1.48–2.30, unadjusted *p* < 0.01, adjusted *p* = 0.02) and comorbidity-polypharmacy scores (OR = 1.30, 95%CI = 0.96–1.85, unadjusted *p* = 0.01, adjusted *p* = 0.047) were associated with significantly increased odds of moderate and increasing complaints (versus mild and decreasing). Greater pTau/Aβ_42_ ratio (OR = 1.56, 95%CI = 0.96–2.24, unadjusted *p* = 0.03, adjusted *p* = 0.08) was also associated with significantly increased odds of moderate and increasing complaints (versus mild and decreasing), but this association did not survive FDR correction. Within this stratum, the trajectory of cognitive complaints was not associated with baseline anxiety, social network, cortical thickness, APOE genotype, or demographic variables (all unadjusted *p* > 0.24, all adjusted *p* > 0.53).

In participants who showed a steep decline in MMSE scores (Stratum #3), an increase of 1 SD in the baseline comorbidity-polypharmacy score multiplied the odds of being in the “stable moderate complaint” class (versus “stable mild” class) by 1.38 (95%CI = 1.03–1.85, unadjusted *p* = 0.03, adjusted *p* = 0.32). However, this association did not survive FDR correction. Within this stratum, the trajectory of cognitive complaints was not associated with the baseline level of AD biomarkers, depression, anxiety, social network, or demographic variables (all unadjusted *p* > 0.09, all adjusted *p* > 0.32).

## Discussion

### Summary of the main findings

Our study contributes to the existing literature by highlighting the main cognitive trajectories in individuals at risk for dementia who have sought medical advice from specialized memory clinics. The majority had, on average, high levels of cognitive performance at baseline that improved slightly over 5 years. Approximately 25% of the sample showed a subtle cognitive decline. Throughout the entire follow-up period, their cognitive performance remained, on average, within normal ranges (mean MMSE > 24 at all visits) but a statistically significant decline was observed. Finally, 15% of the sample suffered a steep cognitive decline during the follow-up.

To our knowledge, this is the first study to examine multiple correlates of subjective cognitive complaint trajectories, while also considering the concurrent objective cognitive trajectory. Although individuals within each stratum showed similar objective cognitive trajectory over time, they exhibited two distinct patterns of subjective cognitive complaints. The odds of expressing one of these patterns of complaints was associated with different correlates depending on objective cognitive trajectory.

Participants with high baseline and slightly increasing MMSE scores were more likely to express moderate and increasing complaints (versus mild and decreasing) if they had higher baseline comorbidity-polypharmacy scores, as well as higher depression scores and fewer close social relationships. The associations between complaint pattern and depression and social relationships did not survive correction for multiple comparisons.

In those with subtle decline in MMSE scores, the odds of moderate and increasing complaints were associated with higher baseline depression and comorbidity-polypharmacy scores, as well as with higher baseline blood-based markers of AD pathology. Only the association between complaint pattern and depression survived correction for multiple comparisons.

Lastly, in participants with a steep decline in MMSE scores, cognitive complaints exhibited two main patterns: either stable moderate or stable mild. The odds of expressing a stable moderate complaint (as opposed to stable mild) were solely associated with higher comorbidity-polypharmacy scores. However, this association did not survive correction for multiple comparisons.

### Comparison with previous literature

The objective cognitive trajectories we have identified offer valuable insights into the natural progression of cognitive change in older adults referred to memory clinics for cognitive complaints or mild impairment. Previous studies [34–36] have found heterogeneous MMSE trajectories, but in individuals with more severe cognitive impairment than in our sample or in community-dwelling elderly. To our knowledge, no other studies with a similar sample composition and approach have shown such heterogeneous cognitive trajectories. Participants who experienced a steep decline in MMSE scores over 5 years were the least numerous (approximately 15%). Almost all individuals in this group had mild impairment at baseline, as evidenced by a CDR of 0.5, and this group had the highest incidence rate of dementia. The subtle cognitive decline observed in approximately 25% of our sample may represent a normal part of the aging process [37] or may reflect a transitional stage between normal cognition and clinically detectable cognitive impairment [38, 39]. It is noteworthy that most of the participants showed consistently high levels of cognitive performance and even improved slightly over time. This observed pattern might be attributed to the practice effect of repeated MMSE administration every 6 months [40]. Familiarity with the assessment process and the potentially positive effects of seeking medical help and receiving reassurance may also contribute to the observed improvement in MMSE scores [41]. While we acknowledge the potential effects of practice in the “subtle decline” and “steep decline” strata, we posit that these effects might be mitigated due to the observed greater cognitive impairment in these strata, which could reduce susceptibility to practice effects. Finally, regarding the identification of three distinct trajectories, we acknowledge that it is plausible that our three trajectories could represent different stages of cognitive change. For example, with a longer follow-up period, individuals in Stratum #2 might end up exhibiting a similar cognitive trajectory as individuals in Stratum #3. However, in our specific sample and follow-up period, our modeling identified three separate groups, each characterized by sufficiently distinct trajectories.

Subjective cognitive complaints and objective cognitive abilities may exhibit a discrepancy at a given time point or follow distinct trajectories over time [19]. Our study contributes by specifically identifying the main trajectories that subjective complaints follow in individuals with a given objective cognitive trajectory, and investigating their correlates.

In all three strata, an initial analysis revealed a significant association between the complaint trajectory and the comorbidity-polypharmacy score. After applying correction for multiple comparisons, this association remained significant only in Stratum #1. The prevalence of comorbid conditions has increased in older adults as mortality rates decrease and the population ages [42]. They may directly affect cognitive abilities and lead to cognitive complaints, possibly through neurochemical imbalances, inflammation, metabolic abnormalities, or side effects of medications [43, 44]. The presence of concomitant diseases may also cause worry, social isolation, or financial stress, which in turn may lead to more severe cognitive complaints [45]. Additionally, cognitive complaints, comorbidity, and polypharmacy have common risk factors such as age or a sedentary lifestyle [46, 47].

The complaint pattern was also associated with depression, particularly in participants with high and slightly increasing MMSE scores (Stratum #1) and in those exhibiting a subtle decline in MMSE scores (Stratum #2). However, upon applying FDR correction, this association remained significant only in Stratum #2. Depressive symptoms are common in the elderly [48, 49] and their nature is complex and not fully understood. Depression can manifest with cognitive slowing or reduced executive functioning [50], aspects that are not comprehensively assessed by the MMSE [22]. In addition, depression has been linked to an increased self-focus and awareness of internal states, often associated with abnormal activation of the Default Mode Network (DMN), a brain network involved in self-reflection and introspection [51]. Consequently, individuals with depression may perceive themselves as cognitively less capable, increasing the likelihood that they will seek medical advice for cognitive complaints. Alternatively, depression may occur in response to self-perception of cognitive changes in both individuals with normal brain aging and brain pathology [52]. This, in turn, may amplify the severity of cognitive complaints.

The lack of association between anxiety and complaint trajectory in this study contrasts with previous findings [53]. One possible explanation for this discrepancy could be the use of a multivariate approach, which may attenuate the specific influence of anxiety on complaint patterns.

Our study also initially suggested an association between loneliness and cognitive complaints in cognitively normal individuals [12, 54]. Participants with fewer close connections were more likely to express moderate and increasing complaints despite otherwise normal cognition. It is noteworthy that, after applying FDR correction, this association did not reach statistical significance. Variability in social support and loneliness in late life has been reported [55–57]. Loneliness and social isolation have been linked to increased stress and inflammation, which can have negative effects on cognitive function [58]. Social isolation may also limit cognitive stimulation and social interaction, crucial for maintaining cognitive health [59]. It is also possible that cognitive complaints themselves lead to social withdrawal, creating a potential bidirectional relationship.

Interestingly, in our initial analysis, an association between complaints pattern and blood pTau/Aβ_42_ ratio was observed exclusively in participants showing a subtle decline in MMSE scores. In selecting the pTau/Aβ42 ratio as a biomarker of AD pathology, we were guided by several compelling reasons. While other studies have questioned the additional value of blood-based AD biomarkers in predicting dementia risk [60], extensive previous research has demonstrated the predictive power of the blood pTau/Aβ42 ratio for early cognitive change and amyloid status in PET scans. Indeed, this ratio is of particular importance as it reflects the balance between beta-amyloid and p-Tau accumulation [25]. Since it is able to capture dynamic changes in AD pathology over time, we considered it particularly suitable for our longitudinal study design. Although it is not currently recommended to measure these biomarkers without objective impairment, they could be employed in the future during cognitive screening appointments [61, 62]. It is noteworthy, however, that after applying the FDR correction, the observed association between the complaint pattern and the blood pTau/Aβ42 ratio no longer showed statistical significance. Although our results initially suggest that moderate and increasing cognitive complaints may reflect initial pathological changes in cognitive function, further investigation is warranted. In individuals with a steeper cognitive decline, the association between complaints and blood biomarkers appears to be overshadowed by their higher average biomarker levels.

Unlike previous studies [63–65], we did not find an association between complaint trajectory and cortical atrophy or genetic susceptibility (i.e., APOE genotype). This highlights the complex and multifactorial nature of cognitive complaints in relation to AD and emphasizes the need for further research on underlying mechanisms and contributing factors.

While our analysis reveals changes in cognitive complaints over time in some groups (such as moderate baseline and increasing complaints, and mild baseline and decreasing complaints), it is important to acknowledge that these observed changes may not necessarily translate into substantial clinical significance, as evidenced by the relatively small differences. An interesting observation emerges regarding the pattern of complaints in individuals with a steep decline in MMSE scores. These individuals had either moderate or mild complaints at baseline that remained stable over time (i.e., no statistically significant difference over time), although their cognitive abilities declined sharply. The concept of the “petrified-self” [66] may provide a framework for understanding this finding. It describes individuals who maintain a stable pattern of cognitive complaints despite apparent decline. This stable sense of self is related to anosognosia [20, 67]. Anosognosia can hinder accurate self-assessment of cognitive decline, posing challenges for healthcare professionals. Recent research has shown that awareness of cognitive decline gradually decreases as the disease progresses, and impaired self-awareness may occur with the first symptoms [17]. Family members, caregivers, and other observers have been shown to be more accurate sources of information than self-reports, especially in advanced stages [68].

### Strengths and limitations

Our study has several strengths. The data were collected as part of a large longitudinal memory clinic-based cohort study. The sample size and 5-year follow-up allowed for a latent class approach to identify different trajectories of both objective cognitive performance and subjective complaints. The carefully selected cohort provided a well-phenotyped and thoroughly assessed sample, enhancing the accuracy and reliability of our findings. Finally, considering multiple factors simultaneously provided a comprehensive understanding of the different factors that may explain the complaint pattern.

A limitation concerns the lack of prior formal validation of the complaint scale used, which could lead to uncertainties regarding its psychometric properties. However, the items are conceptually relevant based on similarity to well-known and validated questionnaires such as the Everyday Cognition (also known as E-Cog [69]) and the Cognitive Function Instrument [70]. In addition, the selection of factors examined in relation to complaint patterns was based on previous literature, clinical experience, and data availability in the MEMENTO. Other factors, such as personality traits [71], would also have been of interest. Regarding biomarkers of AD pathology, we intentionally chose not to examine multiple biomarkers. This deliberate decision was made to reduce the risk of statistical inflation and data mining. By focusing on a single biomarker ratio with robust theoretical and empirical support, we ensured the integrity and interpretability of the results of our study. Although exclusion of certain factors may limit the breadth of the study, the selected factors still provide meaningful information and contribute to knowledge about complaint patterns. Finally, we used a listwise deletion approach that excluded participants with missing data on any of the variables of interest. The challenge of identifying appropriate variables for imputation was a key factor in our decision. While this approach helped maintain the integrity of our longitudinal modeling and minimized the potential bias associated with imputation, it resulted in a smaller sample size. Having a large initial sample size allowed us to maintain a substantial dataset for meaningful statistical analysis.

### Concluding remarks

The present study has shown that, despite a similar objective cognitive trajectory, there is heterogeneity in subjective perceptions of these cognitive changes by those affected. This perception was explained by both AD-related and, more robustly, non-AD-related factors. These findings challenge the prevailing view in research on cognitive complaints, which considers them a major risk factor for dementia and views SCD as a phase preceding MCI in the natural history of AD [72]. We argue for a more comprehensive and holistic approach to the study of cognitive complaints. An overarching goal should be to understand whether particular complaints indicate an underlying AD (e.g., difficulty absorbing new information) and to distinguish them from complaints attributable to other contextual factors (e.g., misplaced keys might be more indicative of attentional problems). In this way, it may be possible to develop effective screening questionnaires for clinical practice that aid in the differentiation and early detection of cognitive disorders.

### Supplementary Information

**Additional file 1.** Full list of the MEMENTO Study Group.

## Data Availability

This work was undertaken using resources on the Dementia Platform UK (DPUK). The Medical Research Council supports DPUK through grant MR/L0237844/2. Any bona fide researcher can apply to access the data via the DPUK Data Access application form (https://portal.dementiasplatform.uk/Apply).
