# Supplementary material for: Understanding factors associated with the trajectory of subjective cognitive complaints in groups with similar objective cognitive trajectories
Source: Alzheimers Res Ther. 2023 Nov 22;15:205. doi: 10.1186/s13195-023-01348-w (PMC10666380; doi:10.1186/s13195-023-01348-w)
Supplement: Supplementary file 1 — Additional file 1. Full list of the MEMENTO Study Group. [file 13195_2023_1348_MOESM1_ESM.pdf]

Supplementary material

Table S1. Complete list of Memento Study Group

Name	Degree	Location	Role
Michèle Allard	MD, PhD	Memory Resource and Research Centre of Bordeaux, CHU de Bordeaux, Hôpital Xavier Arnozan, F-33000, Bordeaux, France	Co-investigator
Sandrine Andrieu	MD, PhD	Memory Resource and Research Centre of Toulouse, CHU de Toulouse, Hôpital La Grave-Casselardit, F-31000, Toulouse, France	Co-investigator
Pierre Anthony	MD, PhD	Memory Resource and Research Centre of Colmar, Hôpitaux Civils de Colmar, F-68000, Colmar, France	Co-investigator
Christine Astier	MD	Memory Resource and Research Centre of Strasbourg, Hôpitaux Universitaires de Strasbourg, F-67000, Strasbourg, France	Co-investigator
Alexandre Augier	MD, PhD	Memory Clinic, Hôpital Avicenne, AP-HP, Hôpitaux Universitaires Paris-Seine-Saint-Denis, F-93009, Bobigny, France	Co-investigator
Nicolas Auguste	MD	Memory Resource and Research Centre of Saint-Etienne, CHU de Saint-Etienne, Hôpital de la Charité, F-42000, Saint-Etienne, France	Co-investigator
Sophie Auriacom be	MD, PhD	Memory Resource and Research Centre of Bordeaux, CHU de Bordeaux, Hôpital Pellegrin, F-33000, Bordeaux, France	Co-investigator
John Avet	MD, PhD	Memory Resource and Research Centre of Saint-Etienne, CHU de Saint-Etienne, Hôpital Nord, F-42000, Saint-Etienne, France	Co-investigator
Olivier Bailon	MD, PhD	Memory Clinic, Hôpital Avicenne, AP-HP, Hôpitaux Universitaires Paris-Seine-Saint-Denis, F-93009, Bobigny, France	Co-investigator
Fabrice-Guy Barral	MD	Memory Resource and Research Centre of Saint-Etienne, CHU de Saint-Etienne, Hôpital Nord, F-42000, Saint-Etienne, France	Co-investigator
Jean Barré	MD	Memory Resource and Research Centre of Angers, CHU d'Angers, F-49000, Angers	Co-investigator
Annick Barthelaix	MD, PhD	Memory Resource and Research Centre of Angers, CHU d'Angers, F-49000, Angers	Co-investigator
Catherine Bayle	MD	Memory Resource and Research Centre of Paris Broca, AP-HP, Paris, France	Co-investigator
Olivier Beauchet		Memory Resource and Research Centre of Angers, CHU d'Angers, F-49000, Angers	Co-investigator
Catherine Belin	MD, PhD	Memory Clinic, Hôpital Avicenne, AP-HP, Hôpitaux Universitaires Paris-Seine-Saint-Denis, F-93009, Bobigny, France	Co-investigator
Samia Belkacem	MD	Institute of Memory and Alzheimer's Disease (IM2A), Centre for NeuroImaging Research (CENIR), Brain and Spine Institute (ICM), UMR S 1127, Department of Neurology, AP-HP, Pitié-Salpêtrière University Hospital, Sorbonne Universities, Pierre et Marie Curie University, Paris, France	Co-investigator
Douraied Ben Salem	MD, PhD	Memory Resource and Research Centre of Brest, CHRU de Brest, F-29000, Brest, France	Co-investigator
Karim Bennys	MD	Memory Resource and Research Centre of Montpellier, CHU de Montpellier, Hôpital Gui de Chauliac, F-34000, Montpellier, France	Co-investigator
Géraldine Bera	MD	Laboratoire d'Imagerie Biomédicale, Sorbonne Universités, UPMC Univ Paris 06, Inserm U1146, CNRS UMR 7371, France NeuroSpin, I2BM, Commissariat à l'Energie Atomique, Paris, France	Co-investigator
Eric Berger	MD	Memory Resource and Research Centre of Besançon, CHU de Besançon, Hôpital Jean Minjoz, Hôpital Saint-Jacques, F-25000, Besançon, France	Co-investigator
Marc G Berger	MD, PhD	Memory Resource and Research Centre of Clermont-Ferrand, CHU de Clermont-Ferrand, F-63000, Clermont-Ferrand, France	Co-investigator
Emilie Bergouin	MD	Memory Resource and Research Centre of Dijon, CHU Dijon Bourgogne, Hôpital du Bocage, Hôpital de Champmaillot, F-21000, Dijon, France	Co-investigator
François Bertin-Hugault	MD	Memory Resource and Research Centre of Lyon, Hospices Civils de Lyon, Hôpital des Charpennes, F-69000, Lyon, France	Co-investigator
Guillaume Bertrand	MD	Memory Clinic, Hôpital Avicenne, AP-HP, Hôpitaux Universitaires Paris-Seine-Saint-Denis, F-93009, Bobigny, France	Co-investigator
François-Xavier Bertrand	MD, PhD	Memory Resource and Research Centre of Nantes, CHU de Nantes, F-44000, Nantes, France	Co-investigator
Catherine Beze	MD	Memory Resource and Research Centre of Center Region, CHRU de Tours, Hôpital Bretonneau, F-37000, Tours, France	Co-investigator
Valérie Boilet		Coordinating Centre, Inserm CIC-1401 Clinical Epidemiology, CHU de Bordeaux, F-33000, Bordeaux, France	Co-investigator
Stéphanie Bombois	MD, PhD	Memory Resource and Research Centre of Lille, CHRU de Lille, Hôpital Roger Salengro, F-59000, Lille, France	Co-investigator
Alain Bonafé	MD, PhD	Memory Resource and Research Centre of Montpellier, CHU de Montpellier, Montpellier, France	Co-investigator

Yasmina Boudali	MD	Memory Resource and Research Centre of Paris Broca, AP-HP, Paris, France	Co-investigator
Hatem Bouhladour	MD, PhD	Memory Resource and Research Centre of Besançon, CHU de Besançon, Hôpital Jean Minjoz, Hôpital Saint-Jacques, F-25000, Besançon, France	Co-investigator
Clémence Boully	MD	Memory Resource and Research Centre of Paris Broca, AP-HP, Paris, France	Co-investigator
Isabelle Bourdel-Marchasson	MD, PhD	Memory Resource and Research Centre of Bordeaux, CHU de Bordeaux, Hôpital Xavier Arnozan, F-33000, Bordeaux, France	Co-investigator
Vincent Bouteloup	PharmD	Coordinating Centre, Inserm CIC-1401 Clinical Epidemiology, CHU de Bordeaux, F-33000, Bordeaux, France	Co-investigator
Claire Boutet	MD	Institute of Memory and Alzheimer's Disease (IM2A), Centre for NeuroImaging Research (CENIR), Brain and Spine Institute (ICM), UMR S 1127, Department of Neurology, AP-HP, Pitié-Salpêtrière University Hospital, Sorbonne Universities, Pierre et Marie Curie University, Paris, France	Co-investigator
Serge Bracard	MD, PhD	Memory Resource and Research Centre of Nancy, CHU de Nancy, F-54000, Nancy, France	Co-investigator
Antoine Brangier	MD	Memory Resource and Research Centre of Angers, CHU d'Angers, F-49000, Angers	Co-investigator
Pierre-Yves Brillet	MD, PhD	Memory Clinic, Hôpital Avicenne, AP-HP, Hôpitaux Universitaires Paris-Seine-Saint-Denis, F-93009, Bobigny, France	Co-investigator
Laure Caillard	MD	Memory Resource and Research Centre of Paris Broca, AP-HP, Paris, France	Co-investigator
Fabienne Calvas	MD	Memory Resource and Research Centre of Toulouse, CHU de Toulouse, Hôpital Purpan, F-31000, Toulouse, France	Co-investigator
Agnès Camus	MD	Memory Resource and Research Centre of Dijon, CHU Dijon Bourgogne, Hôpital du Bocage, Hôpital de Champmaillot, F-21000, Dijon, France	Co-investigator
Vincent Camus	MD, PhD	Memory Resource and Research Centre of Center Region, CHRU de Tours, Hôpital Bretonneau, F-37000, Tours, France	Co-investigator
Sandrine Canaple	MD	Memory Resource and Research of Amiens, CHU Amiens Picardie, F-80000, Amiens, France	Co-investigator
Antoine Carpentier	MD, PhD	Memory Clinic, Hôpital Avicenne, AP-HP, Hôpitaux Universitaires Paris-Seine-Saint-Denis, F-93009, Bobigny, France	Co-investigator
Pascaline Cassagnaud	MD	Memory Resource and Research Centre of Lille, CHRU de Lille, Hôpital Roger Salengro, F-59000, Lille, France	Co-investigator
Françoise Cattin	MD	Memory Resource and Research Centre of Besançon, CHU de Besançon, Hôpital Jean Minjoz, Hôpital Saint-Jacques, F-25000, Besançon, France	Co-investigator
Ludivine Chamard	MD	Memory Resource and Research Centre of Besançon, CHU de Besançon, Hôpital Jean Minjoz, Hôpital Saint-Jacques, F-25000, Besançon, France	Co-investigator
Stéphane Chanalet	MD	Memory Resource and Research Centre of Nice, CHU de Nice, Hôpital Pasteur, F-06100, Nice, France	Co-investigator
Mathieu Chastan	MD	Memory Resource and Research Centre of Rouen, CLCC Henri Becquerel, Rouen, France	Co-investigator
Sophie Chauvelier	MD	Memory Resource and Research Centre of Paris Broca, AP-HP, Paris, France	Co-investigator
Valérie Chauvire	MD	Memory Resource and Research Centre of Angers, CHU d'Angers, F-49000, Angers	Co-investigator
Samia Cheriet	MD, PhD	Memory Resource and Research Centre of Toulouse, CHU de Toulouse, Hôpital Purpan, F-31000, Toulouse, France	Co-investigator
Anthony Clotagatide	MD	Memory Resource and Research Centre of Saint-Etienne, CHU de Saint-Etienne, Hôpital Nord, F-42000, Saint-Etienne, France	Co-investigator
Emmanuel Cognat	MD, PhD	Memory Resource and Research Centre of Paris Nord, AP-HP, Paris, France	Co-investigator
Lora Cohen	PhD	Memory Resource and Research Centre of Grenoble, CHU de Grenoble Alpes, Grenoble, France	Co-investigator
Jean-Marc Constans	MD, PhD	Memory Resource and Research of Amiens, CHU Amiens Picardie, F-80000, Amiens, France	Co-investigator
Marie-Hélène Coste	MD, PhD	Memory Resource and Research Centre of Lyon, Hospices Civils de Lyon, Hôpital des Charpennes, F-69000, Lyon, France	Co-investigator
Jean-Philippe Cottier	MD, PhD	Memory Resource and Research Centre of Center Region, CHRU de Tours, Hôpital Bretonneau, F-37000, Tours, France	Co-investigator
François Cotton	MD, PhD	Memory Resource and Research Centre of Lyon, Hospices Civils de Lyon, Hôpital des Charpennes, F-69000, Lyon, France	Co-investigator
Isabelle Couret	MD	Memory Resource and Research Centre of Montpellier, CHU de Montpellier, Hôpital Gui de Chauliac, F-34000, Montpellier, France	Co-investigator

Olivier-François Couturier	MD, PhD	Memory Resource and Research Centre of Angers, CHU d’Angers, F-49000, Angers	Co-investigator
Pascale Cowppli-Bony	MD, PhD	Memory Resource and Research Centre of Bordeaux, CHU de Bordeaux, Hôpital Pellegrin, F-33000, Bordeaux, France	Co-investigator
Véronique Cressot	MD	Memory Resource and Research Centre of Bordeaux, CHU de Bordeaux, Hôpital Xavier Arnozan, F-33000, Bordeaux, France	Co-investigator
Benjamin Crétin	MD	Memory Resource and Research Centre of Strasbourg, Hôpitaux Universitaires de Strasbourg, F-67000, Strasbourg, France	Co-investigator
Keren Danaila	MD	Memory Resource and Research Centre of Lyon, Hospices Civils de Lyon, Hôpital des Charpennes, F-69000, Lyon, France	Co-investigator
Jacques Darcourt	MD, PhD	Memory Resource and Research Centre of Nice, CLCC Antoine Lacassagne, Nice, France	Co-investigator
Jean-François Dartigues	MD, PhD	Memory Resource and Research Centre of Bordeaux, CHU de Bordeaux, Hôpital Pellegrin, F-33000, Bordeaux, France	Co-investigator
Ana-Maria Dascalita	MD, PhD	Memory Resource and Research Centre of Saint-Etienne, CHU de Saint-Etienne, Hôpital de la Charité, F-42000, Saint-Etienne, France	Co-investigator
Renaud David	MD, PhD	Memory Resource and Research Centre of Nice, CHU de Nice, Institut Claude Pompidou, F-06100, Nice, France	Co-investigator
Xavier De Petigny	MD	Memory Resource and Research Centre of Strasbourg, Hôpitaux Universitaires de Strasbourg, F-67000, Strasbourg, France	Co-investigator
Delphine De Verbizier-Lonjon	MD	Memory Resource and Research Centre of Montpellier, CHU de Montpellier, Hôpital Gui de Chauliac, F-34000, Montpellier, France	Co-investigator
Marielle Decousus	MD, PhD	Memory Resource and Research Centre of Saint-Etienne, CHU de Saint-Etienne, Hôpital Nord, F-42000, Saint-Etienne, France	Co-investigator
Isabelle Defouilloy	MD, PhD	Memory Resource and Research of Amiens, CHU Amiens Picardie, F-80000, Amiens, France	Co-investigator
Christine Delmaire	MD, PhD	Memory Resource and Research Centre of Lille, CHRU de Lille, Hôpital Roger Salengro, F-59000, Lille, France	Co-investigator
Julien Delrieu	MD	Memory Resource and Research Centre of Toulouse, CHU de Toulouse, Hôpital La Grave-Casselardit, F-31000, Toulouse, France	Co-investigator
Catherine Demuyinck	MD	Memory Resource and Research Centre of Strasbourg, Hôpitaux Universitaires de Strasbourg, F-67000, Strasbourg, France	Co-investigator
Vincent Deramecourt	MD, PhD	Memory Resource and Research Centre of Lille, CHRU de Lille, Hôpital Roger Salengro, F-59000, Lille, France	Co-investigator
Hervé Deramond	MD, PhD	Memory Resource and Research of Amiens, CHU Amiens Picardie, F-80000, Amiens, France	Co-investigator
Thomas Desmidt	MD, PhD	Memory Resource and Research Centre of Center Region, CHRU de Tours, Hôpital Bretonneau, F-37000, Tours, France	Co-investigator
Marie-Dominique Desruet	PharmD, PhD	Memory Resource and Research Centre of Grenoble, CHU de Grenoble Alpes, Grenoble, France	Co-investigator
Julien Detour		Memory Resource and Research Centre of Strasbourg, Hôpitaux Universitaires de Strasbourg, F-67000, Strasbourg, France	Co-investigator
Agnès Devendeville	MD	Memory Resource and Research of Amiens, CHU Amiens Picardie, F-80000, Amiens, France	Co-investigator
Mira Didic	MD, PhD	Memory Resource and Research Centre of Marseille, CHU de Marseille, Hôpital La Timone, F-13000, Marseille, France	Co-investigator
Maritchu Doireau	MD	Memory Resource and Research Centre of Bordeaux, CHU de Bordeaux, Hôpital Pellegrin, F-33000, Bordeaux, France	Co-investigator
Antonio Dos Santos	MD	Institute of Memory and Alzheimer's Disease (IM2A), Brain and Spine Institute (ICM), UMR S 1127, Department of Neurology, AP-HP, Pitié-Salpêtrière University Hospital, Sorbonne Universities, Pierre et Marie Curie University, Paris, France	Co-investigator
Patrice Douillet	MD	Memory Resource and Research Centre of Montpellier, CHU de Montpellier, Hôpital Gui de Chauliac, F-34000, Montpellier, France	Co-investigator
Foucaud Du Boisgueheneuc	MD	Memory Resource and Research Centre of Poitiers, CHU de Poitiers, Hôpital de La Milétrie, F-86000, Poitiers, France	Co-investigator
Delphine Dubail	MD	Memory Resource and Research Centre of Paris Broca, AP-HP, Paris, France	Co-investigator
Laure Ducroq-Ducastaing	MD	Memory Resource and Research Centre of Brest, CHRU de Brest, F-29000, Brest, France	Co-investigator
Julien Dumurgier	MD, PhD	Memory Resource and Research Centre of Paris Nord, AP-HP, Paris, France	Co-investigator

Diane Dupuy	MD, PhD	Memory Resource and Research of Amiens, CHU Amiens Picardie, F-80000, Amiens, France	Co-investigator
Emmanuel le Duron	MD, PhD	Memory Resource and Research Centre of Paris Broca, AP-HP, Paris, France	Co-investigator
Inna Dygai-Cochet	MD, PhD	Memory Resource and Research Centre of Dijon, CLCC Georges François Leclerc, Dijon, France	Co-investigator
Véronique Eder	MD, PhD	Memory Clinic, Hôpital Avicenne, AP-HP, Hôpitaux Universitaires Paris-Seine-Saint-Denis, F-93009, Bobigny, France	Co-investigator
Stéphane Epelbaum	MD, PhD	Institute of Memory and Alzheimer's Disease (IM2A), Brain and Spine Institute (ICM), UMR S 1127, Department of Neurology, AP-HP, Pitié-Salpêtrière University Hospital, Sorbonne Universities, Pierre et Marie Curie University, Paris, France	Co-investigator
Frédérique Etcharry-Bouyx	MD, PhD	Memory Resource and Research Centre of Angers, CHU d'Angers, F-49000, Angers	Co-investigator
Daniel Fagret	MD, PhD	Memory Resource and Research Centre of Grenoble, CHU de Grenoble Alpes, Grenoble, France	Co-investigator
Catherine Faisant	MD	Memory Resource and Research Centre of Toulouse, CHU de Toulouse, Hôpital La Grave-Casselardit, F-31000, Toulouse, France	Co-investigator
Karim Farid	MD, PhD	Memory Resource and Research Centre of Paris Nord, AP-HP, Paris, France	Co-investigator
Denis Fédérico	MD	Memory Resource and Research Centre of Lyon, Hospices Civils de Lyon, Hôpital des Charpennes, F-69000, Lyon, France	Co-investigator
Olivier Felician	MD, PhD	Memory Resource and Research Centre of Marseille, CHU de Marseille, Hôpital La Timone, F-13000, Marseille, France	Co-investigator
Philippe Fernandez	MD, PhD	Memory Resource and Research Centre of Bordeaux, CHU de Bordeaux, Hôpital Pellegrin, F-33000, Bordeaux, France	Co-investigator
Pacôme Fosse	MD	Memory Resource and Research Centre of Angers, CHU d'Angers, F-49000, Angers	Co-investigator
Alexandra Foubert-Samier	MD, PhD	Memory Resource and Research Centre of Bordeaux, CHU de Bordeaux, Hôpital Pellegrin, F-33000, Bordeaux, France	Co-investigator
Isabelle Franck	MD	Memory Resource and Research Centre of Strasbourg, Hôpitaux Universitaires de Strasbourg, F-67000, Strasbourg, France	Co-investigator
Monique Galitzky	MD	Memory Resource and Research Centre of Toulouse, CHU de Toulouse, Hôpital Purpan, F-31000, Toulouse, France	Co-investigator
Céline Gallazzini-Crepin	MD	Memory Resource and Research Centre of Grenoble, CHU de Grenoble Alpes, Grenoble, France	Co-investigator
Radka Gantchev	MD	Memory Resource and Research Centre of Marseille, CHU de Marseille, Hôpital La Timone, F-13000, Marseille, France	Co-investigator
Laurence Garbarg-Chenon	MD	Memory Clinic, Hôpital Avicenne, AP-HP, Hôpitaux Universitaires Paris-Seine-Saint-Denis, F-93009, Bobigny, France	Co-investigator
Guillaume Gautier	MD, PhD	Memory Resource and Research Centre of Marseille, CHU de Marseille, Hôpital La Timone, F-13000, Marseille, France	Co-investigator
Emmanuel Gerardin	MD, PhD	Memory Resource and Research Centre of Rouen, Neuroradiology Department, Rouen University Hospital, F-76031, Rouen, France	Co-investigator
Claire Gervais	MD	Memory Resource and Research Centre of Nice, CHU de Nice, Institut Claude Pompidou, F-06100, Nice, France	Co-investigator
Jean-Claude Getenet	MD	Memory Resource and Research Centre of Saint-Etienne, CHU de Saint-Etienne, Hôpital Nord, F-42000, Saint-Etienne, France	Co-investigator
Nadine Girard	MD, PhD	Memory Resource and Research Centre of Marseille, CHU de Marseille, Hôpital La Timone, F-13000, Marseille, France	Co-investigator
Fabienne Giraud	MD	Memory Resource and Research Centre of Marseille, CHU de Marseille, Hôpital La Timone, F-13000, Marseille, France	Co-investigator
Chantal Girtanner	MD	Memory Resource and Research Centre of Saint-Etienne, CHU de Saint-Etienne, Hôpital de la Charité, F-42000, Saint-Etienne, France	Co-investigator
Valérie Gissot	MD	Memory Resource and Research Centre of Center Region, CHRU de Tours, Hôpital Bretonneau, F-37000, Tours, France	Co-investigator
Caroline Grangeon	PharmD	Memory Resource and Research Centre of Nice, CHU de Nice, Institut Claude Pompidou, F-06100, Nice, France	Co-investigator
Daniel Grucker	MD, PhD	Memory Resource and Research Centre of Strasbourg, Hôpitaux Universitaires de Strasbourg, F-67000, Strasbourg, France	Co-investigator
Eric Guedj	MD, PhD	Memory Resource and Research Centre of Marseille, CHU de Marseille, Hôpital La Timone, F-13000, Marseille, France	Co-investigator
Claude Gueriot	MD	Memory Resource and Research Centre of Marseille, CHU de Marseille, Hôpital La Timone, F-13000, Marseille, France	Co-investigator
Yves Guilhermet	MD	Memory Resource and Research Centre of Lyon, Hospices Civils de Lyon, Hôpital des Charpennes, F-69000, Lyon, France	Co-investigator
Rémy Guillevin	MD, PhD	Memory Resource and Research Centre of Poitiers, CHU de Poitiers, Hôpital de La Milétrie, F-86000, Poitiers, France	Co-investigator

Sophie Haffen	MD	Memory Resource and Research Centre of Besançon, CHU de Besançon, Hôpital Jean Minjoz, Hôpital Saint-Jacques, F-25000, Besançon, France	Co-investigator
Didier Hannequin	MD, PhD	Memory Resource and Research Centre of Rouen, Neurology Department, Rouen University Hospital, F-76031, Rouen, France	Co-investigator
Sandrine Harston	MD	Memory Resource and Research Centre of Bordeaux, CHU de Bordeaux, Hôpital Xavier Arnozan, F-33000, Bordeaux, France	Co-investigator
Anne Hitzel	MD, PhD	Memory Resource and Research Centre of Toulouse, CHU de Toulouse, Hôpital Purpan, F-31000, Toulouse, France	Co-investigator
Caroline Hommet	MD, PhD	Memory Resource and Research Centre of Center Region, CHRU de Tours, Hôpital Bretonneau, F-37000, Tours, France	Co-investigator
Claude Hossein-Foucher	MD, PhD	Memory Resource and Research Centre of Lille, CHRU de Lille, Hôpital Roger Salengro, F-59000, Lille, France	Co-investigator
Fabrice Hubele	MD	Memory Resource and Research Centre of Strasbourg, Hôpitaux Universitaires de Strasbourg, F-67000, Strasbourg, France	Co-investigator
Agnès Jacquin-Piques	MD, PhD	Memory Resource and Research Centre of Dijon, CHU Dijon Bourgogne, Hôpital du Bocage, Hôpital de Champmaillot, F-21000, Dijon, France	Co-investigator
Betty Jean	MD	Memory Resource and Research Centre of Clermont-Ferrand, CHU de Clermont-Ferrand, F-63000, Clermont-Ferrand, France	Co-investigator
Joanne Jenn	MD, PhD	Memory Resource and Research Centre of Bordeaux, CHU de Bordeaux, Hôpital Xavier Arnozan, F-33000, Bordeaux, France	Co-investigator
Laure Joly	MD, PhD	Memory Resource and Research Centre of Nancy, CHU de Nancy, F-54000, Nancy, France	Co-investigator
Thérèse Jonveaux	MD	Memory Resource and Research Centre of Nancy, CHU de Nancy, F-54000, Nancy, France	Co-investigator
Adrien Julian	MD, PhD	Memory Resource and Research Centre of Poitiers, CHU de Poitiers, Hôpital de La Milétrie, F-86000, Poitiers, France	Co-investigator
Aurélie Kas	MD, PhD	Laboratoire d'Imagerie Biomédicale, Sorbonne Universités, UPMC Univ Paris 06, Inserm U1146, CNRS UMR 7371, France NeuroSpin, I2BM, Commissariat à l'Energie Atomique, Paris, France	Co-investigator
Anna Kearney-Schwartz	MD	Memory Resource and Research Centre of Nancy, CHU de Nancy, F-54000, Nancy, France	Co-investigator
Alice Keles	MD	Memory Resource and Research Centre of Nancy, CHU de Nancy, F-54000, Nancy, France	Co-investigator
Antony Kelly	MD	Memory Resource and Research Centre of Clermont-Ferrand, Centre de Lutte contre le Cancer, F-63000, Clermont-Ferrand, France	Co-investigator
Nathalie Keromnes	MD	Memory Resource and Research Centre of Brest, CHRU de Brest, F-29000, Brest, France	Co-investigator
Lejla Koric	MD	Memory Resource and Research Centre of Marseille, CHU de Marseille, Hôpital La Timone, F-13000, Marseille, France	Co-investigator
Alexandre Krainik	MD, PhD	Memory Resource and Research Centre of Grenoble, CHU de Grenoble Alpes, Grenoble, France	Co-investigator
Stéphane Kremer	MD	Memory Resource and Research Centre of Strasbourg, Hôpitaux Universitaires de Strasbourg, F-67000, Strasbourg, France	Co-investigator
Florian Labourée	MD	Memory Resource and Research Centre of Paris Broca, AP-HP, Paris, France	Co-investigator
Franck Lacoeuille	MD, PhD	Memory Resource and Research Centre of Angers, CHU d’Angers, F-49000, Angers	Co-investigator
Francoise Lala	MD	Memory Resource and Research Centre of Toulouse, CHU de Toulouse, Hôpital La Grave-Casselardit, F-31000, Toulouse, France	Co-investigator
Chantal Lamy	MD	Memory Resource and Research of Amiens, CHU Amiens Picardie, F-80000, Amiens, France	Co-investigator
Jean-Louis Laplanche	PharmD, PhD	Memory Resource and Research Centre of Paris Nord, AP-HP, Paris, France	Co-investigator
Cyrille Launay	MD, PhD	Memory Resource and Research Centre of Angers, CHU d’Angers, F-49000, Angers	Co-investigator
Stéphane Lehericy	MD, PhD	Institute of Memory and Alzheimer's Disease (IM2A), Centre for NeuroImaging Research (CENIR), Brain and Spine Institute (ICM), UMR S 1127, Department of Neurology, AP-HP, Pitié-Salpêtrière University Hospital, Sorbonne Universities, Pierre et Marie Curie University, Paris, France	Co-investigator
Sylvain Lehmann	MD, PhD	Memory Resource and Research Centre of Montpellier, CHU de Montpellier, Hôpital Gui de Chauliac, F-34000, Montpellier, France	Co-investigator
Hermine Lenoir	MD, PhD	Memory Resource and Research Centre of Paris Broca, AP-HP, Paris, France	Co-investigator
Marcel Levy	MD, PhD	Institute of Memory and Alzheimer's Disease (IM2A), Brain and Spine Institute (ICM), UMR S 1127, Department of Neurology, AP-HP, Pitié-Salpêtrière University Hospital, Sorbonne Universities, Pierre et Marie Curie University, Paris, France	Co-investigator
Stéphanie Libercier	MD, PhD	Memory Resource and Research Centre of Colmar, Hôpitaux Civils de Colmar, F-68000, Colmar, France	Co-investigator

Marie-Anne Mackowiak-Cordoliani	MD	Memory Resource and Research Centre of Lille, CHRU de Lille, Hôpital Roger Salengro, F-59000, Lille, France	Co-investigator
Eloi Magnin	MD	Memory Resource and Research Centre of Besançon, CHU de Besançon, Hôpital Jean Minjoz, Hôpital Saint-Jacques, F-25000, Besançon, France	Co-investigator
Zaza Makaroff	MD	Memory Resource and Research Centre of Lyon, Hospices Civils de Lyon, Hôpital des Charpennes, F-69000, Lyon, France	Co-investigator
Athina Marantidou	MD	Memory Clinic, Hôpital Avicenne, AP-HP, Hôpitaux Universitaires Paris-Seine-Saint-Denis, F-93009, Bobigny, France	Co-investigator
Isabelle Marcet	MD	Memory Resource and Research Centre of Bordeaux, CHU de Bordeaux, Hôpital Pellegrin, F-33000, Bordeaux, France	Co-investigator
Cécilia Marelli	MD, PhD	Memory Resource and Research Centre of Montpellier, CHU de Montpellier, Hôpital Gui de Chauliac, F-34000, Montpellier, France	Co-investigator
Sophie Marilier	MD	Memory Resource and Research Centre of Dijon, CHU Dijon Bourgogne, Hôpital du Bocage, Hôpital de Champmaillot, F-21000, Dijon, France	Co-investigator
Idalie Martin	MD	Memory Resource and Research Centre of Lyon, Hospices Civils de Lyon, Hôpital des Charpennes, F-69000, Lyon, France	Co-investigator
Olivier Martinaud	MD, PhD	Memory Resource and Research Centre of Rouen, Neurology Department, Rouen University Hospital, F-76031, Rouen, France	Co-investigator
Catherine Martin-Hunyadi	MD	Memory Resource and Research Centre of Strasbourg, Hôpitaux Universitaires de Strasbourg, F-67000, Strasbourg, France	Co-investigator
Aïcha Medjoul	MD	Memory Clinic, Hôpital Avicenne, AP-HP, Hôpitaux Universitaires Paris-Seine-Saint-Denis, F-93009, Bobigny, France	Co-investigator
Isabelle Merlet	MD	Memory Resource and Research Centre of Poitiers, CHU de Poitiers, Hôpital de La Milétrie, F-86000, Poitiers, France	Co-investigator
Danielle Mestas	MD	Memory Resource and Research Centre of Clermont-Ferrand, CHU de Clermont-Ferrand, F-63000, Clermont-Ferrand, France	Co-investigator
Marc-Etienne Meyer	MD, PhD	Memory Resource and Research of Amiens, CHU Amiens Picardie, F-80000, Amiens, France	Co-investigator
Jean-Marc Michel	MD	Memory Resource and Research Centre of Colmar, Hôpitaux Civils de Colmar, F-68000, Colmar, France	Co-investigator
Agnès Michon	MD	Institute of Memory and Alzheimer's Disease (IM2A), Brain and Spine Institute (ICM), UMR S 1127, Department of Neurology, AP-HP, Pitié-Salpêtrière University Hospital, Sorbonne Universities, Pierre et Marie Curie University, Paris, France	Co-investigator
Isabelle Migeon-Duballet	MD	Memory Resource and Research Centre of Poitiers, CHU de Poitiers, Hôpital de La Milétrie, F-86000, Poitiers, France	Co-investigator
Karl Mondon	MD, PhD	Memory Resource and Research Centre of Center Region, CHRU de Tours, Hôpital Bretonneau, F-37000, Tours, France	Co-investigator
Clément Morgat	PharmD, PhD	Memory Resource and Research Centre of Bordeaux, CHU de Bordeaux, Hôpital Pellegrin, F-33000, Bordeaux, France	Co-investigator
Véronique Moullart	MD	Memory Resource and Research of Amiens, CHU Amiens Picardie, F-80000, Amiens, France	Co-investigator
Christian Moussard	MD	Memory Resource and Research Centre of Besançon, CHU de Besançon, Hôpital Jean Minjoz, Hôpital Saint-Jacques, F-25000, Besançon, France	Co-investigator
Aurélie Mouton	MD, PhD	Memory Resource and Research Centre of Nice, CHU de Nice, Institut Claude Pompidou, F-06100, Nice, France	Co-investigator
Izzie Jacques Namer	MD, PhD	Memory Resource and Research Centre of Strasbourg, Hôpitaux Universitaires de Strasbourg, F-67000, Strasbourg, France	Co-investigator
Georges Niewiadomski	MD, PhD	Memory Resource and Research Centre of Nice, CHU de Nice, Institut Claude Pompidou, F-06100, Nice, France	Co-investigator
Guillaume Nivaggioni	MD	Memory Resource and Research Centre of Nice, CHU de Nice, Institut Claude Pompidou, F-06100, Nice, France	Co-investigator
Marie Noblet	MD, PhD	Memory Resource and Research Centre of Strasbourg, Hôpitaux Universitaires de Strasbourg, F-67000, Strasbourg, France	Co-investigator
Michel Nonent	MD, PhD	Memory Resource and Research Centre of Brest, CHRU de Brest, F-29000, Brest, France	Co-investigator
Fati Nourhashemi	MD, PhD	Memory Resource and Research Centre of Toulouse, CHU de Toulouse, Hôpital La Grave-Casselardit, F-31000, Toulouse, France	Co-investigator
Hélène Oesterle	MD	Memory Resource and Research Centre of Colmar, Hôpitaux Civils de Colmar, F-68000, Colmar, France	Co-investigator
Galdric Orvoen	MD	Memory Resource and Research Centre of Paris Broca, AP-HP, Paris, France	Co-investigator
Pierre Jean Ousset	MD, PhD	Memory Resource and Research Centre of Toulouse, CHU de Toulouse, Hôpital La Grave-Casselardit, F-31000, Toulouse, France	Co-investigator

Amandine Pallardy	MD	Memory Resource and Research Centre of Nantes, CHU de Nantes, F-44000, Nantes, France	Co-investigator
Claire Paquet	MD, PhD	Memory Resource and Research Centre of Paris Nord, AP-HP, Paris, France	Co-investigator
Pierre-Yves Pare	MD, PhD	Memory Resource and Research Centre of Angers, CHU d'Angers, F-49000, Angers	Co-investigator
Anne Pasco	MD, PhD	Memory Resource and Research Centre of Angers, CHU d'Angers, F-49000, Angers	Co-investigator
Pierre Payoux	MD, PhD	Memory Resource and Research Centre of Toulouse, CHU de Toulouse, Hôpital Purpan, F-31000, Toulouse, France	Co-investigator
Cécile Pays	MD, PhD	Memory Resource and Research Centre of Montpellier, CHU de Montpellier, Hôpital Gui de Chauliac, F-34000, Montpellier, France	Co-investigator
Isabelle Pellegrin	MD, PhD	Biological Research Centre, CHU de Bordeaux, F-33000, Bordeaux, France	Co-investigator
Rémy Perdrisot	MD, PhD	Memory Resource and Research Centre of Poitiers, CHU de Poitiers, Hôpital de La Milétrie, F-86000, Poitiers, France	Co-investigator
Bertille Perin	MD, PhD	Memory Resource and Research of Amiens, CHU Amiens Picardie, F-80000, Amiens, France	Co-investigator
Christine Perret-Guillaume	MD, PhD	Memory Resource and Research Centre of Nancy, CHU de Nancy, F-54000, Nancy, France	Co-investigator
Grégory Petyt	MD	Memory Resource and Research Centre of Lille, CHRU de Lille, Hôpital Roger Salengro, F-59000, Lille, France	Co-investigator
Nathalie Philippi	MD, PhD	Memory Resource and Research Centre of Strasbourg, Hôpitaux Universitaires de Strasbourg, F-67000, Strasbourg, France	Co-investigator
Geneviève Pinganaud	MD	Memory Resource and Research Centre of Bordeaux, CHU de Bordeaux, Hôpital Xavier Arnozan, F-33000, Bordeaux, France	Co-investigator
Matthieu Plichart	MD	Memory Resource and Research Centre of Paris Broca, AP-HP, Paris, France	Co-investigator
Gabriel Pop	MD, PhD	Memory Clinic, Hôpital Avicenne, AP-HP, Hôpitaux Universitaires Paris-Seine-Saint-Denis, F-93009, Bobigny, France	Co-investigator
Michèle Puel	MD	Memory Resource and Research Centre of Toulouse, CHU de Toulouse, Hôpital Purpan, F-31000, Toulouse, France	Co-investigator
Mathieu Queneau	MD, PhD	Memory Resource and Research Centre of Paris Nord, Centre Cardiologique du Nord, Paris, France	Co-investigator
Solène Querellou	MD	Memory Resource and Research Centre of Brest, CHRU de Brest, F-29000, Brest, France	Co-investigator
Muriel Quillard-Muraine	MD, PhD	Memory Resource and Research Centre of Rouen, Neurology Department, Rouen University Hospital, F-76031, Rouen, France	Co-investigator
Valérie Quipourt	MD, PhD	Memory Resource and Research Centre of Dijon, CHU Dijon Bourgogne, Hôpital du Bocage, Hôpital de Champmaillot, F-21000, Dijon, France	Co-investigator
Chloé Rachez	MD, PhD	Memory Resource and Research Centre of Clermont-Ferrand, CHU de Clermont-Ferrand, F-63000, Clermont-Ferrand, France	Co-investigator
Micheline Razzouk-Cadet	MD	Memory Resource and Research Centre of Nice, CHU de Nice, Institut Claude Pompidou, F-06100, Nice, France	Co-investigator
Anne-Sophie Rigaud	MD, PhD	Memory Resource and Research Centre of Paris Broca, AP-HP, Paris, France	Co-investigator
Hélène Robin-Ismer	MD	Memory Resource and Research Centre of Strasbourg, Hôpitaux Universitaires de Strasbourg, F-67000, Strasbourg, France	Co-investigator
Mathieu Rodallec	MD, PhD	Memory Resource and Research Centre of Paris Nord, Centre Cardiologique du Nord, Paris, France	Co-investigator
Yves Rolland	MD, PhD	Memory Resource and Research Centre of Toulouse, CHU de Toulouse, Hôpital La Grave-Casselardit, F-31000, Toulouse, France	Co-investigator
Adeline Rollin-Sillaire	MD, PhD	Memory Resource and Research Centre of Lille, CHRU de Lille, Hôpital Roger Salengro, F-59000, Lille, France	Co-investigator
Olivier Rouaud	MD	Memory Resource and Research Centre of Dijon, CHU Dijon Bourgogne, Hôpital du Bocage, Hôpital de Champmaillot, F-21000, Dijon, France	Co-investigator
Caroline Roubaud	MD, PhD	Memory Resource and Research Centre of Lyon, Hospices Civils de Lyon, Hôpital des Charpennes, F-69000, Lyon, France	Co-investigator
Isabelle Rouch	MD, PhD	Memory Resource and Research Centre of Lyon, Hospices Civils de Lyon, Hôpital des Charpennes, F-69000, Lyon, France	Co-investigator
Julie Roux	MD, PhD	Memory Resource and Research Centre of Grenoble, CHU de Grenoble Alpes, Grenoble, France	Co-investigator
Guillaume Sacco	MD, PhD	Memory Resource and Research Centre of Nice, CHU de Nice, Institut Claude Pompidou, F-06100, Nice, France	Co-investigator
Pierre-Yves Salaun	MD	Memory Resource and Research Centre of Brest, CHRU de Brest, F-29000, Brest, France	Co-investigator

François Salmon	MD, PhD	Memory Resource and Research Centre of Poitiers, CHU de Poitiers, Hôpital de La Milétrie, F-86000, Poitiers, France	Co-investigator
Alicia Sanchez	MD	Memory Resource and Research Centre of Saint-Etienne, CHU de Saint-Etienne, Hôpital Nord, F-42000, Saint-Etienne, France	Co-investigator
Maria-Joao Santiago-Ribeiro	MD, PhD	Memory Resource and Research Centre of Center Region, CHRU de Tours, Hôpital Bretonneau, F-37000, Tours, France	Co-investigator
Alain Sarciron	MD	Memory Resource and Research Centre of Lyon, Hospices Civils de Lyon, Hôpital des Charpennes, F-69000, Lyon, France	Co-investigator
Nathalie Sastre-Hengan	MD	Memory Resource and Research Centre of Toulouse, CHU de Toulouse, Hôpital La Grave-Casselardit, F-31000, Toulouse, France	Co-investigator
Mathilde Sauvée	MD, PhD	Memory Resource and Research Centre of Grenoble, CHU de Grenoble Alpes, Grenoble, France	Co-investigator
Christian Scheiber	MD, PhD	Memory Resource and Research Centre of Lyon, Hospices Civils de Lyon, Hôpital des Charpennes, F-69000, Lyon, France	Co-investigator
Anne-Marie Schneider	MD, PhD	Memory Resource and Research Centre of Strasbourg, Hôpitaux Universitaires de Strasbourg, F-67000, Strasbourg, France	Co-investigator
Franck Semah	MD, PhD	Memory Resource and Research Centre of Lille, CHRU de Lille, Hôpital Roger Salengro, F-59000, Lille, France	Co-investigator
Amélie Serra	MD	Memory Resource and Research Centre of Grenoble, CHU de Grenoble Alpes, Grenoble, France	Co-investigator
Marie-Laure Seux	MD	Memory Resource and Research Centre of Paris Broca, AP-HP, Paris, France	Co-investigator
Hélène Sordet-Guépet	MD	Memory Resource and Research Centre of Dijon, CHU Dijon Bourgogne, Hôpital du Bocage, Hôpital de Champmaillot, F-21000, Dijon, France	Co-investigator
Maria Eugenia Soto	MD	Memory Resource and Research Centre of Toulouse, CHU de Toulouse, Hôpital La Grave-Casselardit, F-31000, Toulouse, France	Co-investigator
Mathieu Tafani	MD	Memory Resource and Research Centre of Toulouse, CHU de Toulouse, Hôpital Purpan, F-31000, Toulouse, France	Co-investigator
Jean-Yves Tanguy	MD, PhD	Memory Resource and Research Centre of Angers, CHU d'Angers, F-49000, Angers	Co-investigator
Michael Taroux	MD, PhD	Memory Resource and Research Centre of Dijon, CHU Dijon Bourgogne, Hôpital du Bocage, Hôpital de Champmaillot, F-21000, Dijon, France	Co-investigator
Marc Teichmann	MD, PhD	Institute of Memory and Alzheimer's Disease (IM2A), Brain and Spine Institute (ICM), UMR S 1127, Department of Neurology, AP-HP, Pitié-Salpêtrière University Hospital, Sorbonne Universities, Pierre et Marie Curie University, Paris, France	Co-investigator
Catherine Terrat	MD, PhD	Memory Resource and Research Centre of Saint-Etienne, CHU de Saint-Etienne, Hôpital de la Charité, F-42000, Saint-Etienne, France	Co-investigator
Jamila Thabet	MD	Memory Clinic, Hôpital Avicenne, AP-HP, Hôpitaux Universitaires Paris-Seine-Saint-Denis, F-93009, Bobigny, France	Co-investigator
Claire Thalamas	MD	Memory Resource and Research Centre of Toulouse, CHU de Toulouse, Hôpital Purpan, F-31000, Toulouse, France	Co-investigator
Catherine Thomas-Anterion	MD, PhD	Memory Resource and Research Centre of Saint-Etienne, CHU de Saint-Etienne, Hôpital Nord, F-42000, Saint-Etienne, France	Co-investigator
Anne-Cécile Troussière	MD	Memory Resource and Research Centre of Lille, CHRU de Lille, Hôpital Roger Salengro, F-59000, Lille, France	Co-investigator
Renata Ursu	MD	Memory Clinic, Hôpital Avicenne, AP-HP, Hôpitaux Universitaires Paris-Seine-Saint-Denis, F-93009, Bobigny, France	Co-investigator
Pierre Vera	MD, PhD	Memory Resource and Research Centre of Rouen, CLCC Henri Becquerel, Rouen, France	Co-investigator
Martine Vercelletto	MD	Memory Resource and Research Centre of Nantes, CHU de Nantes, F-44000, Nantes, France	Co-investigator
Olivier Vercruysse	MD	Memory Resource and Research Centre of Lille, CHRU de Lille, Hôpital Roger Salengro, F-59000, Lille, France	Co-investigator
Antoine Verger	MD, PhD	Memory Resource and Research Centre of Nancy, CHU de Nancy, F-54000, Nancy, France	Co-investigator
Philippe Viau	MD	Memory Resource and Research Centre of Nice, CHU de Nice, Institut Claude Pompidou, F-06100, Nice, France	Co-investigator
Marie-Neige Videau	MD	Memory Resource and Research Centre of Bordeaux, CHU de Bordeaux, Hôpital Xavier Arnozan, F-33000, Bordeaux, France	Co-investigator
Thierry Voisin	MD	Memory Resource and Research Centre of Toulouse, CHU de Toulouse, Hôpital La Grave-Casselardit, F-31000, Toulouse, France	Co-investigator

Nathalie Wagemann	MD, PhD	Memory Resource and Research Centre of Nantes, CHU de Nantes, F-44000, Nantes, France	Co-investigator
Aziza Waissi-Sediq	MD	Memory Resource and Research Centre of Lyon, Hospices Civils de Lyon, Hôpital des Charpennes, F-69000, Lyon, France	Co-investigator
Jing Xie	MD, PhD	Memory Resource and Research Centre of Lyon, Hospices Civils de Lyon, Hôpital des Charpennes, F-69000, Lyon, France	Co-investigator
Nathanaëlle Yeni	MD	Laboratoire d'Imagerie Biomédicale, Sorbonne Universités, UPMC Univ Paris 06, Inserm U1146, CNRS UMR 7371, France NeuroSpin, I2BM, Commissariat à l'Energie Atomique, Paris, France	Co-investigator
Michel Zanca	MD, PhD	Memory Resource and Research Centre of Montpellier, CHU de Montpellier, Hôpital Gui de Chauliac, F-34000, Montpellier, France	Co-investigator
Jean Zinszner	MD, PhD	Memory Clinic, Hôpital Avicenne, AP-HP, Hôpitaux Universitaires Paris-Seine-Saint-Denis, F-93009, Bobigny, France	Co-investigator

Table S2. Description of the variables of interest within the three strata of objective cognitive trajectory in our subsample of MEMENTO study participants.

Stratum	**Stratum #1** ***High and increasing MMSE*** **(n=1,044)**	**Stratum #2** ***Subtle decline in MMSE scores*** **(n=445)**	**Stratum #3** ***Steep decrease in MMSE scores*** **(n=259)**
Blood pTau/Aβ_{42} at baseline [pg/mL; mean (SD)]	0.10 (0.23)	0.11 (0.13)	0.17 (0.21)
Cortical thickness at baseline [cm^3; mean (SD)]	2.35 (0.10)	2.33 (0.10)	2.28 (0.09)
APOE genotype [Noncarrier; n (%)]	795 (76.1)	248 (55.7)	130 (50.2)
Comorbidity-polypharmacy at baseline [mean (SD)]	6.68 (4.57)	8.02 (4.94)	7.03 (4.34)
Depression score at baseline [mean (SD)]	1.19 (2.55)	1.67 (3.29)	1.82 (3.11)
Anxiety score at baseline [mean (SD)]	1.61 (3.10)	1.97 (3.10)	2.42 (3.93)
Social relationships [Nb of close relationships; mean (SD)]	6.38 (5.41)	6.03 (6.99)	6.73 (6.21)

Note. Numerical variables are described by means and SD (standard deviations). Categorical variables by counts and percentages. Low education: less than a high school diploma. pTau/Aβ_{42}: ratio between levels of threonine$_{181}$ pTau and β-amyloid$_{42}$. APOE: Apolipoprotein E.
